# Association between LDL/HDL ratio and hypertension in Chinese middle-aged and older adults: a cross-sectional and longitudinal analysis based on CHARLS LDL/HDL ration and hypertension

**DOI:** 10.3389/fendo.2025.1484318

**Published:** 2025-02-14

**Authors:** Weicheng Lai, Xiao Chen, Lerui Wang, Liangxiu Wu, Xin Li, Boda Zhou

**Affiliations:** ^1^ Department of Cardiology, Nanjing BenQ Medical Center, The Affiliated BenQ Hospital of Nanjing Medical University, Nanjing, Jiangsu, China; ^2^ Department of Cardiology, Beijing Tsinghua Changgung Hospital, School of Clinical Medicine, Tsinghua University, Beijing, China; ^3^ School of Clinical Medicine, Tsinghua University, Beijing, China; ^4^ Department of Gastroenterology, The People’s Hospital of Hezhou, Hezhou, Guangxi, China

**Keywords:** CHARLS, hypertension, LDL/HDL ratio, prevalence, incidence

## Abstract

**Introduction:**

Hypertension is a global public health issue and major risk factor for cardiovascular disease (CVD). Low-density lipoprotein/high-density lipoprotein ratio (LDL/HDL Ratio, LHR) is an important indicator of lipid metabolism related to CVD. However, the relationship between LHR and the prevalence and incidence of hypertension has not been reported in large populations. This study aims to investigate the association between LHR and hypertension in middle-aged and elderly population.

**Methods:**

This study utilized the China Health and Retirement Longitudinal Study (CHARLS) database from 2011 to 2020. Cross-sectional study was employed to analyze the association between LHR and the prevalence of hypertension; longitudinal analysis was used to examine the association between LHR and the incidence of hypertension. Eligible participants were adults aged 45 years and older with complete LHR and self-reported hypertension records. Multivariate logistic regression, smooth curve fitting, threshold effect analysis was performed.

**Results:**

In the cross-sectional study, we included 13,150 participants. After adjusting for potential confounders, each one-unit increase in LHR was associated with a 22% increase in the prevalence of hypertension (OR = 1.22, 95% CI: 1.15-1.30, *P* < 0.0001). The association between LHR and hypertension was consistent across different subgroups, with higher LHR being more strongly associated with increased hypertension prevalence in females and non-smokers. Our results revealed a linear relationship between LHR and hypertension prevalence. Longitudinal analysis showed that, among participants without hypertension in 2011, after 7 years of follow-up, the association between LHR and hypertension incidence remained robust after adjusting for a wide range of demographic, clinical, and biochemical variables (*P* < 0.05).

**Conclusions:**

These results demonstrated significant positive association between LHR and the prevalence & incidence of hypertension, in a nationwide representative middle-aged and elderly population in China.

## Introduction

Hypertension is a public health issue and major risk factor for cardiovascular diseases (CVDs), with atherosclerosis as common mechanism (1). Understanding and managing hypertension involves considering the role of lipid metabolism, particularly the balance between low-density lipoprotein cholesterol (LDL-C) and high-density lipoprotein cholesterol (HDL-C). The ratio of LDL-C to HDL-C (LDL/HDL Ratio, LHR) is an important indicator of cardiovascular health and plays a crucial role in the development and progression of hypertension (2, 3).

LHR not only reflects lipid metabolism but also plays a critical role in vascular health. Elevated LHR is associated with atherosclerosis, vascular stiffness, and endothelial dysfunction, all of which contribute to the pathogenesis of hypertension (4). For instance, higher LDL-C levels promote cholesterol deposition in the arterial wall, leading to arterial rigidity and increased vascular resistance, ultimately resulting in elevated blood pressure (5). Meanwhile, lower HDL-C levels impair the body’s ability to clear cholesterol, exacerbating the harmful effects of LDL-C and increasing the risk of hypertension.

Recent studies have demonstrated that LHR is an effective predictor of cardiovascular events (6), manifesting greater predictive value than measuring LDL-C or HDL-C levels alone (7). Specifically, LHR was significantly associated with all-cause mortality in hypertensive patients **≥** 65 years old in China (8). Previous studies have also established a potential mechanism why LHR could impact hypertension, with elevated LDL-C levels leading to endothelial dysfunction, inflammation and oxidative stress, arterial narrowing and hardening, consequently hypertension (9). Meanwhile, lower HDL-C levels attenuated protective effect against these processes (10). However, despite multiple studies establishing the predictive value of LHR in cardiovascular health, there is still a lack of large-scale epidemiological data directly evaluating the relationship between LHR and the prevalence/incidence of hypertension. Given the increasing trend of hypertension in the aging population of China, it is particularly important to further investigate this association.

The aim of this study is to investigate the association between the LHR and the prevalence & incidence of hypertension in middle-aged and older populations. Thus, we conducted a cross-sectional and longitudinal analysis on data from the China Health and Retirement Longitudinal Study (CHARLS), a nationally representative study in 45 years and above population in China. These data allow for an effective assessment of the relationship between LHR and the risk of hypertension in middle-aged and older population, a high-risk population for hypertension.

## Methods

### Study design and population

This study utilized data from the CHARLS, gathered from 150 counties or districts and 450 villages across 28 provinces in China, containing demographic, economic, health status, blood tests and functional information. Baseline survey was performed in 2011 (Wave 1), follow-ups were conducted every two years, Wave 2 in 2013, Wave 3 in 2015, Wave 4 in 2018, Wave 5 in 2020, blood tests were only performed in 2011 and 2015. Access to the CHARLS dataset is available via its official website at charls.ccer.edu.cn/en.

**Figure 1:**
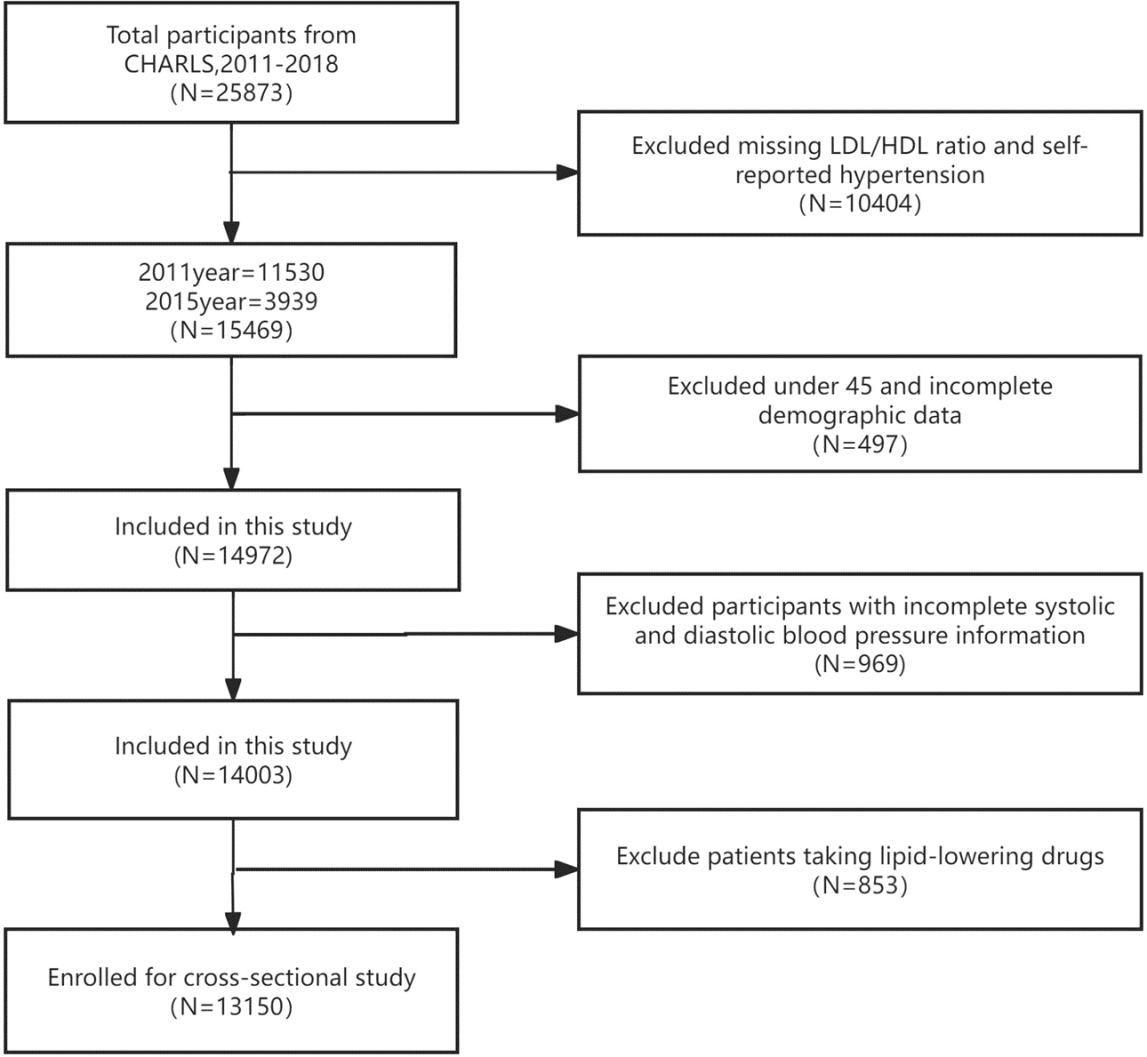
Flowchart of participant selection.

In the cross-sectional analysis, we combined data from Wave 1 and Wave 3. Inclusion criteria were aged 45 years and older; and complete hypertension diagnostic data, DBP, SBP, LDL-C, HDL-C; and complete sociodemographic information. Exclusion criteria were age < 45 years; or incomplete hypertension diagnostic data, DBP, SBP, LDL-C, HDL-C; or incomplete sociodemographic information; or history of lipid-lowering medication use. After rigorous screening, 13,150 participants qualified for the cross-sectional analysis, with flowchart shown in **Figure 1**.

**Figure 2:**
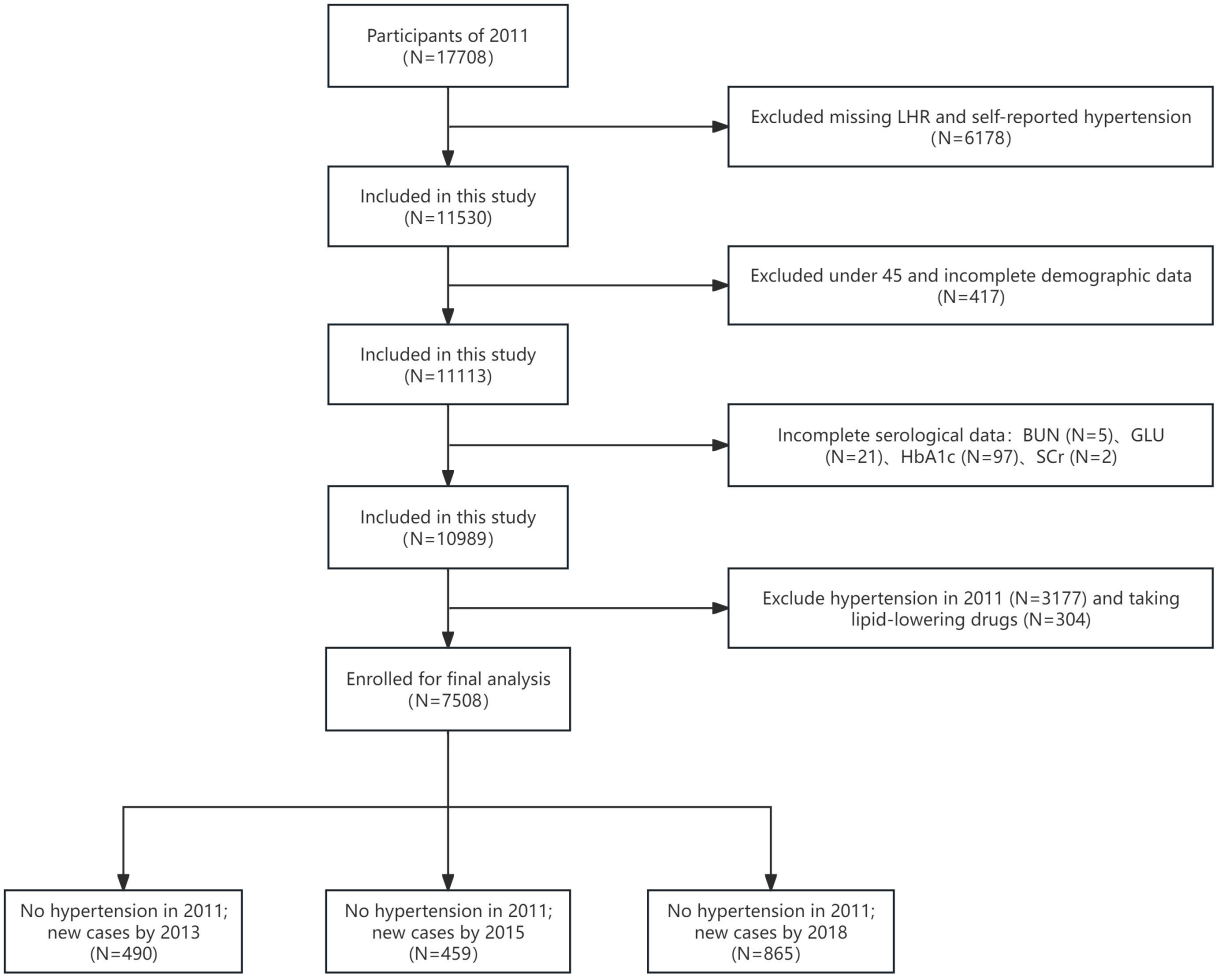
Flowchart of participant selection for longitudinal analysis.

For the longitudinal analysis, we selected participants without hypertension in 2011, focusing on the incidence of hypertension during follow-up. Inclusion criteria were complete LHR value in 2011; and complete hypertension diagnostic data during at least one follow-up. Exclusion criteria were age < 45 years; or incomplete hypertension diagnostic data, DBP, SBP, LDL-C, HDL-C; or incomplete sociodemographic information; or history of lipid-lowering medication use. After rigorous screening, 7,508 participants qualified for the longitudinal analysis, with flowchart shown in **Figure 2**.

### Ethical approval

The CHARLS project and the protocol for biomarker sample collection were approved by the Biomedical Ethics Review Committee of Peking University (IRB00001052-11014) and the Institutional Review Board of the National School of Development at Peking University (IRB00001052-11015), with informed consent obtained from all participants.

### Data collection and potential covariates

Data collection was performed by professionally trained personnel via structured questionnaires to gather sociodemographic information. Health-related behaviors such as smoking and drinking status, medical history (diabetes, heart disease, and stroke), and medication used for diabetes, dyslipidemia or hypertension were also recorded. Trained professionals conducted physical measurements, including height, weight, and blood pressure. Blood pressure was measured using Omron HEM-7200 sphygmomanometer, the average of three readings was recorded.

Fasting venous blood samples were collected in the morning to measure fasting blood glucose (FBG), HbA1c, triglyceride (TG), total cholesterol (TC), HDL-C, LDL-C, serum creatinine (Scr), blood urea nitrogen (BUN), and serum uric acid (SUA) levels.

The covariates included gender, age, education level, smoking status, drinking status, SBP, DBP, BMI, TC, TG, FBG, CRP, HbA1c, Scr, BUN and SUA levels.

### Measurement of LHR

The LHR was calculated (11) by LDL-C/HDL-C, both of which are expressed in mg/dL. In subsequent analysis, we examined LHR as both a continuous variable and categorized it into two distinct groups based on the ROC threshold value (high LHR group: LHR > 2.14; low LHR group: LHR ≤ 2.14) to enhance the analytical strength.

### Definition of hypertension

Hypertension was defined by one of the following criteria (12): ① positive answer to question “Have you ever been diagnosed with hypertension by a doctor?” or ② positive answer to question “Are you currently taking any treatments to manage or control your hypertension, such as Traditional Chinese Medicine or Western modern medicine?”

### Statistical analysis

Statistical analysis was performed via R software (version 4.3.1) and Empower (version 6.0). Continuous variables are expressed as the means ± standard deviations, and categorical variables are expressed as numbers and percentages. Differences in variables across different LHR groups were compared via one-way ANOVA, Kruskal−Wallis H test, or chi−square test. Three models were employed: Model 1 was unadjusted; Model 2 adjusted for gender, age, education level, smoking status, drinking status, SBP, DBP and BMI; Model 3 adjusted for TC, TG, Scr, BUN, CRP, HbA1c, SUA and FBG on basis of Model 2. The association between the LHR and hypertension prevalence/incidence was evaluated, the results were presented as odds ratios (ORs) and 95% confidence intervals (CIs).

A generalized additive model (GAM) was used to explore the nonlinear relationship between LHR and hypertension prevalence, and a segmented regression model was used for threshold effect analysis. Subgroup analyses were conducted on the basis of gender, age, smoking status, drinking status, HbA1c, FBG, BMI, with interactions tested via multivariate logistic regression models. Statistical significance was set at *p* < 0.05.

In the longitudinal analysis, we utilized the 7-year follow-up data from the CHARLS database (2011-2018) to track participants over time. Similar to the cross-sectional study, participants were categorized into two groups based on the ROC of LHR: the low LHR group (LHR ≤ 2.36) and the high LHR group (LHR > 2.36). To assess the long-term impact of LHR on hypertension incidence, we employed a stepwise multivariable logistic regression model and made appropriate adjustments following the principles of cohort studies. This approach allowed us to thoroughly examine the relationship between LHR and hypertension incidence, while accounting for temporal dynamics. The results are presented as ORs with their corresponding 95% CIs, highlighting the long-term effects of LHR on hypertension risk.

## Results

### Baseline characteristics

There were 25,873 participants enrolled in the CHARLS cohort initially. After excluding missing data and lipid-lowering medication use, 13,150 participants were included in the cross-sectional analysis. Participants were divided into two groups based on LHR: Low LHR group (LHR ≤ 2.14, N=6281) and High LHR group (LHR > 2.14, N=6869). The prevalence of hypertension, diabetes, and stroke was significantly higher in High LHR group than in Low LHR group (*p* < 0.001). Additionally, SBP and DBP, and biochemical indicators such as TC, TG, BUN, SUA, CRP, FBG, and HbA1c were significantly higher in High LHR group (*p* < 0.001). The High LHR group had lower rates of smoking and drinking (*p* < 0.001) (**Table 1**).

**Table 1:** Baseline characteristics of participants LHR in cross-sectional study.

Variable	Total	Q1 ≤2.14	Q2 >2.14	*P* value
**Participants, sample size (N)**	13150	6281	6869	***NA***
**Age,years**	59.47 ± 9.48	59.53 ± 9.71	59.40 ± 9.25	0.432
Gender (%)				0.002
Male	6222 (47.38)	3059 (48.70)	3163 (46.05)	
Female	6928 (52.62)	3222 (51.30)	3706 (53.95)	
Education level (%)				<0.001
Below high school or vocational school	11818 (89.92)	5708 (90.89)	6110 (88.95)	
High school or vocational school	1160 (8.79)	510 (8.12)	650 (9.46)	
Above high school or vocational school	171 (1.29)	62 (0.99)	109 (1.59)	
Marital status (%)				<0.001
Married	7620 (79.77)	3680 (78.73)	3940 (80.80)	
Married separation	655 (6.88)	362 (7.74)	293 (6.01)	
Separated	41 (0.44)	28 (0.60)	13 (0.27)	
Divorced	90 (0.94)	44 (0.94)	46 (0.94)	
Widowed	1047 (10.96)	504 (10.78)	543 (11.14)	
Never married	97 (1.02)	56 (1.20)	41 (0.84)	
Hypertension (%)				<0.001
yes	3625 (27.41)	1507 (23.99)	2118 (30.83)	
no	9525 (72.59)	4774 (76.01)	4751 (69.17)	
Diabetes (%)				<0.001
yes	593 (5.00)	209 (3.83)	384 (6.17)	
no	11085 (95.00)	5244 (96.17)	5841 (93.83)	
Dyslipidemia (%)				<0.001
yes	991 (7.46)	356 (5.67)	635 (9.24)	
no	12159 (92.54)	5925 (94.33)	6234 (90.76)	
Stroke (%)				<0.001
yes	257 (2.16)	93 (1.70)	164 (2.62)	
no	11484 (97.84)	5380 (98.30)	6104 (97.38)	
**SBP,mmHg**	128.54 ± 20.85	126.75 ± 20.62	130.33 ± 21.08	<0.001
**DBP,mmHg**	75.11 ± 12.00	74.09 ± 12.11	76.13 ± 11.88	<0.001
Antihypertensive medication (%)				<0.001
yes	2538 (19.41)	1025 (16.58)	1513 (22.23)	
no	10451 (80.60)	5158 (83.42)	5293 (77.77)	
Antidiabetic medication (%)				<0.001
yes	482 (3.64)	186 (2.96)	296 (4.31)	
no	12668 (96.36)	6095 (97.04)	6573 (95.69)	
Drinking (%)				<0.001
yes	4896 (41.85)	2491 (45.39)	2405 (38.31)	
no	6870 (58.15)	2997 (54.61)	3873 (61.69)	
Smoking (%)				<0.001
yes	4726 (40.27)	2292 (41.77)	2434 (38.76)	
no	7040 (59.74)	3195 (58.23)	3845 (61.24)	
BMI (%)				<0.001
<18.5	870 (6.84)	606 (9.77)	264 (3.90)	
18.5-23	5354 (41.62)	3073 (49.56)	2281 (33.67)	
23-25	2668 (20.48)	1153 (18.59)	1515 (22.36)	
≥25	4084 (31.08)	1369 (22.08)	2715 (40.07)	
**TC ,mg/dL**	189.17 ± 34.32	174.68 ± 31.71	203.65 ± 36.93	<0.001
**TG ,mg/dL**	131.89 ± 91.10	116.24 ± 98.59	147.53 ± 83.61	<0.001
**BUN ,mg/dL**	15.67 ± 4.63	15.81 ± 4.79	15.52 ± 4.46	0.002
**SUA ,mg/dL**	4.62 ± 1.33	4.53 ± 1.32	4.70 ± 1.33	<0.001
**Scr ,mg/dL**	0.79 ± 0.25	0.78 ± 0.27	0.80 ± 0.23	<0.001
**CRP ,mg/dL**	2.75 ± 7.21	2.45 ± 6.90	3.04 ± 7.51	<0.001
**FBG ,mmol/L**	5.88 ± 1.73	5.69 ± 1.51	6.06 ± 1.95	<0.001
**HbA1c (%)**	5.48 ± 0.90	5.42 ± 0.80	5.54 ± 1.00	<0.001

Data are presented as mean ± standard deviation or number (%).

NA stands for "Not Applicable".

### Association between LHR and hypertension prevalence

Multivariate regression analysis revealed a significant association between LHR and hypertension prevalence. Each unit increase in LHR was associated with 22% (OR = 1.22, 95% CI: 1.15-1.30, *p* < 0.0001) increase in hypertension prevalence, after adjusting for potential covariates. Specifically, High LHR group was associated with 32% (OR=1.32, 95% CI: 1.18-1.47, *p* < 0.0001) increase in hypertension prevalence, compared with Low LHR group. These results indicate a significant association between higher LHR and increased hypertension prevalence (**Table 2**).

**Table 2:** Multivariate regression analysis of the association between LHR and hypertension prevalence.

Variable	Model 1		Model 2		Model 3	
	OR (95% CI)	*p* value	OR (95% CI)	*p* value	OR (95% CI)	*p* value
**LHR**	1.25 (1.20, 1.30)	<0.0001	1.19 (1.13, 1.25)	<0.0001	1.22 (1.15, 1.30)	<0.0001
**Low LHR ( LHR ≤ 2.14 )**	**Ref.**		**Ref.**		**Ref.**	
**High LHR ( LHR > 2.14 )**	1.41 (1.31, 1.53)	<0.0001	1.31 (1.20, 1.44)	<0.0001	1.32 (1.18, 1.47)	<0.0001

Model 1 adjusted for none. Model 2 adjusted for gender, age,education level, smoking status, drinking status, SBP, DBP, and BMI. Model 3 adjusted for FBG, Scr, BUN, SUA, CRP, HbA1c, TC, TG on the basis of Model 2. LHR as a continuous variable and grouped by ROC threshold.

### Linear relationship between LHR and hypertension

**Figure 3:**
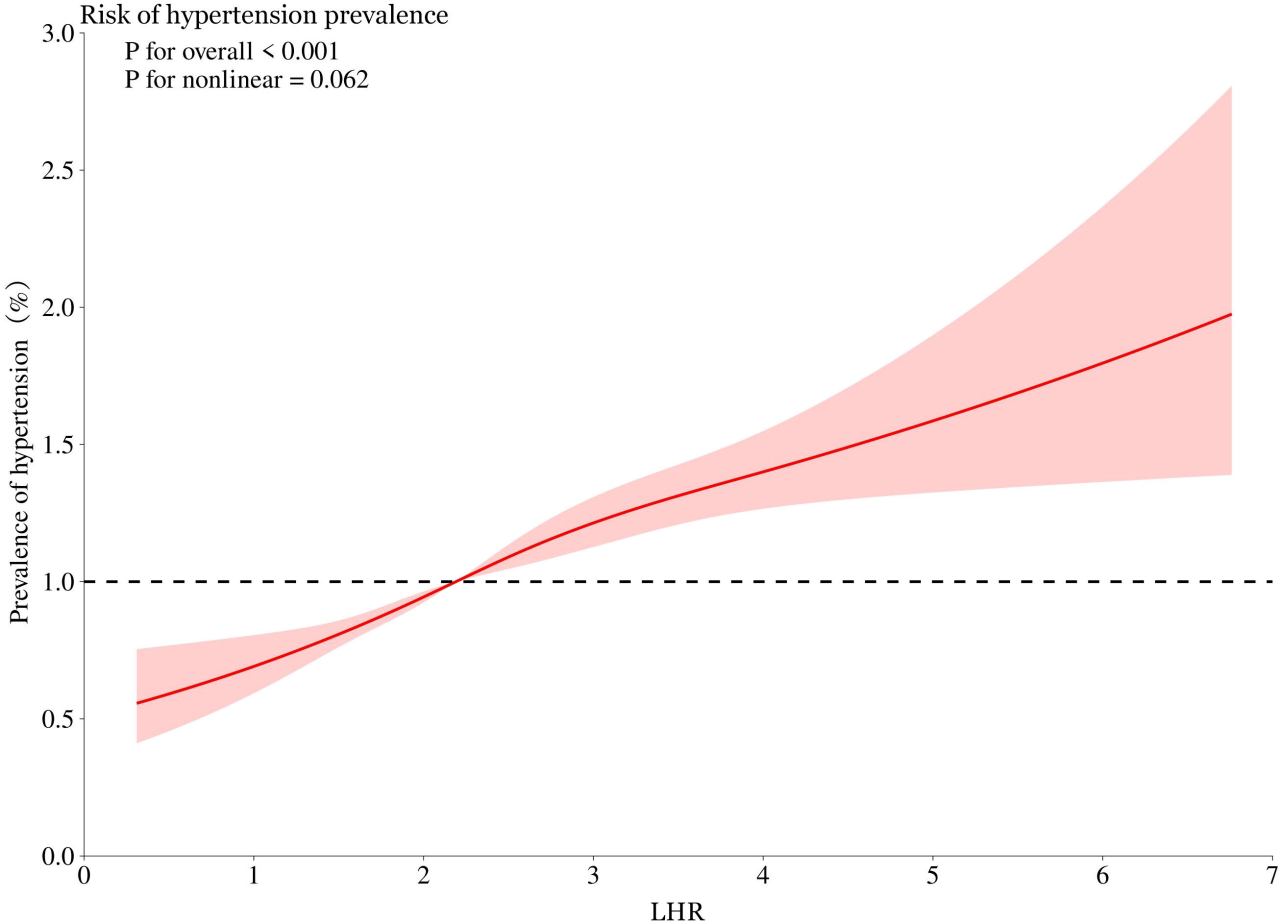
Smooth curve fitting was used to evaluate the linear relationship between LHR and hypertension prevalence. The red solid line represents the probability of hypertension prevalence, and the blue dotted line represents the 95% confidence interval curve. LHR, Low-Density Lipoprotein to High-Density Lipoprotein Ratio.

Using smooth curve fitting analysis, we assessed the association between LHR and hypertension prevalence, as shown in **Figure 3**. The analysis revealed a linear relationship between LHR and hypertension (*p* for non-linearity = 0.062).

### Subgroup analysis

**Figure 4:**
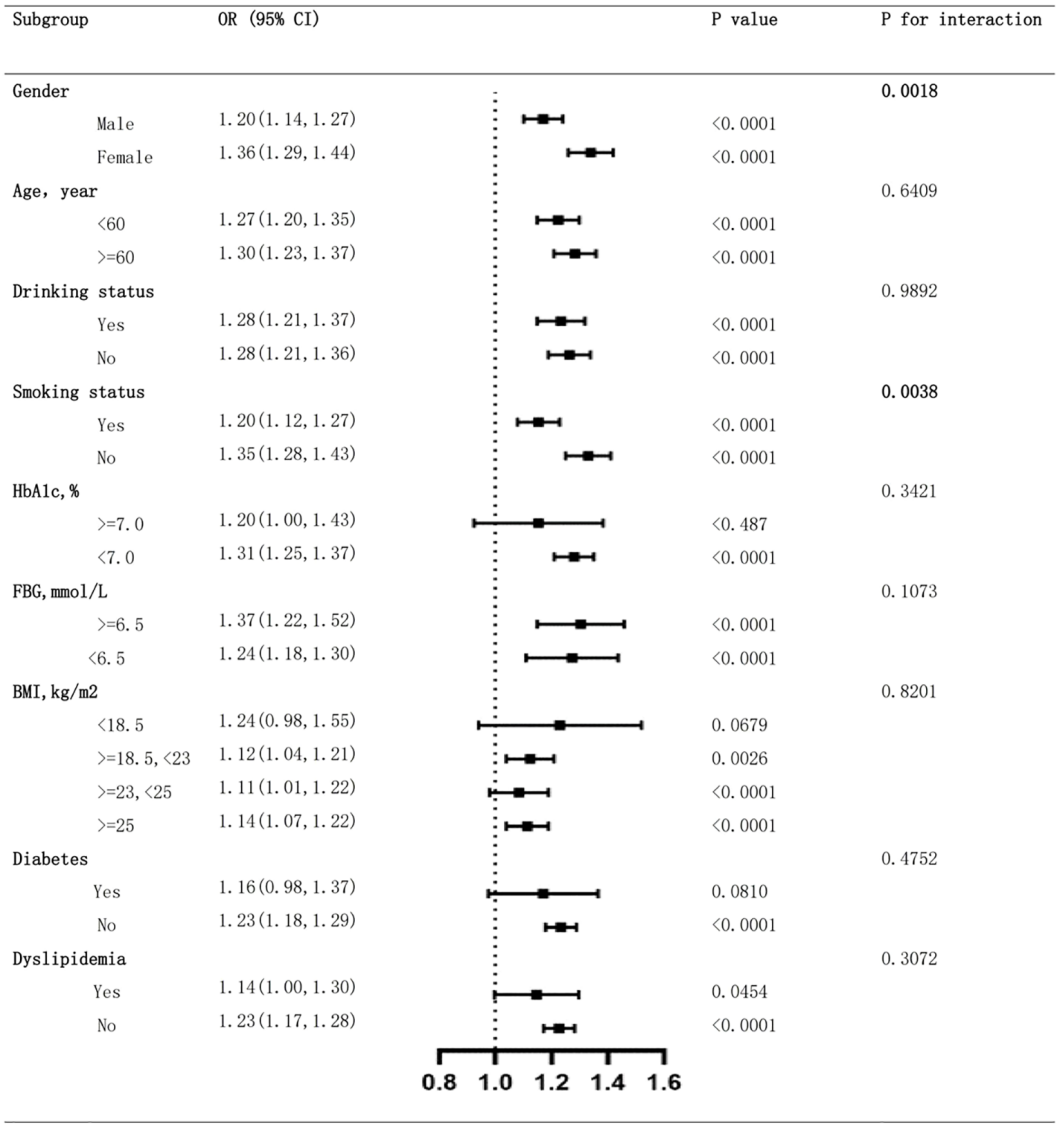
Subgroup analysis of the association between LHR and hypertension prevalence. LHR, Low-Density Lipoprotein to High-Density Lipoprotein Ratio.

To ensure the reliability of our findings, we conducted a series of subgroup analysis to test the consistency of results across different subgroups. As shown in **Figure 4**, except for the subgroups with HbA1c level > 7.0%, or BMI level < 18.5, all other subgroups demonstrated significant association between LHR and the prevalence of hypertension (*p* < 0.05), which could be attributed to limited sample size of these subgroups.

We identified significant interaction between gender and LHR (p for interaction 0.0018), suggesting female participants were more sensitive to LHR for the prevalence of hypertension than male. We also found significant interaction between smoking status and LHR (p for interaction 0.0038), where never smoked participants were more sensitive to LHR than those ever smoked. Further analysis ruled out interactions between LHR and age, drinking status, HbA1c, FBG, BMI, diabetes and dyslipidemia, indicating that these variables did not significantly alter the association between LHR and hypertension prevalence (all p for interaction > 0.05).

### Association between LHR and hypertension incidence

A longitudinal analysis was conducted to explore the relationship between LHR and hypertension incidence over 2-, 4-, and 7-year follow-up periods. When LHR was analyzed as a continuous variable, after adjusting for potential covariates, each one-unit increase in LHR was associated with a 12% increase in hypertension incidence (OR = 1.12, 95% CI: 1.03-1.20, p = 0.0045). These results indicate that LHR is an independent and significant predictor of hypertension incidence. Even after adjusting for a wide range of demographic, clinical, and biochemical variables, the association between LHR and hypertension remained robust (**Table 3**).

**Table 3:** Multivariate regression analysis of the association between LHR and hypertension incidence.

Variable	Model 1		Model 2		Model 3	
	OR (95% CI)	*p* value	OR (95% CI)	*p* value	OR (95% CI)	*p* value
**LHR**	1.14 (1.08, 1.20)	<0.0001	1.13 (1.06, 1.21)	0.0002	1.12 (1.03, 1.20)	0.0045
**Low LHR ( LHR ≤ 2.36 )**	**Ref.**		**Ref.**		**Ref.**	
**High LHR ( LHR > 2.36 )**	1.22 (1.10, 1.36)	0.0002	1.21 (1.07, 1.37)	0.0025	1.15 (1.01, 1.32)	0.0408

Model 1 adjusted for none. Model 2 adjusted for gender, age,education level, smoking status, drinking status, SBP, DBP, and BMI. Model 3 adjusted for FBG, Scr, BUN, SUA, CRP, HbA1c, TC, TG on the basis of Model 2. LDL/HDL Ratio as a continuous variable and grouped by ROC threshold.

## Discussion

This study, which is based on nationally representative data, explored the association between the LHR and hypertension in the Chinese population aged 45 years and above. Our analysis of 13,150 middle-aged and elderly participants revealed a significant association between elevated LHR and hypertension prevalence. Subgroup analysis further confirmed the stability of this positive association. Previous studies have demonstrated that an imbalance in LDL-C and HDL-C levels is a significant risk factor for CVDs (13–15). Our study provides direct evidence of the association between elevated LHR and hypertension prevalence: each unit increase in the LHR was associated with 22% increase in hypertension prevalence.

Subgroup analysis further revealed the variability in the association between the LHR and hypertension risk across gender, age, and lifestyle factors. The association between the LHR and hypertension risk was more pronounced in females and non-smokers, suggesting that special attention should be given to these high-risk subgroups in clinical practice (16). Possible mechanisms may relate to physiological characteristics of lipid metabolism, hormone levels in women (17), as well as non-smoking individuals may have better vascular elasticity and are more sensitive to changes in lipid metabolism (18).

Based on our investigation, no studies to date have specifically examined the relationship between LHR and hypertension incidence. Previous studies have focused primarily on the TG/HDL or TC/HDL Ratios (19, 20). Grover’s (21) model identified LHR as a potential lipid marker for predicting cardiovascular events, with hypertension incidence being indirectly linked to cardiovascular events. The longitudinal analysis further supported the value of the LHR as a predictor of hypertension incidence. Following individuals without hypertension at baseline in 2011, we found that the LHR serves as a stable and continuous predictor of future hypertension development, especially in the middle-aged and elderly population. Timely intervention and adjustment of lipid metabolism may effectively reduce the incidence of hypertension (22).

Our smooth curve fitting analysis indicates a linear relationship between LHR and hypertension prevalence, as shown in **Figure 3**. The non-linearity test yielded a p-value of 0.062, suggesting that the relationship is primarily linear. The clinical significance of this linear relationship is that an increase in LHR is proportionally related to an increase in hypertension risk, highlighting the importance of monitoring and managing LHR levels in high-risk patients (23). While LHR showed strong predictive power in this study, its interactions with traditional cardiovascular risk factors, such as BMI, diabetes status, and family history, warrant further investigation. Future studies should also account for dynamic changes in LHR over time by incorporating repeated measurements, allowing for a more nuanced assessment of its relationship with hypertension prevalence and incidence. This approach would offer a deeper understanding of LHR’s clinical significance.

The strengths of this study include the use of the nationally representative CHARLS database, which covers various sociodemographic and health-related factors, enhancing the generalizability and credibility of the findings. However, this study also has several limitations. Firstly, although we adjusted for several confounding factors, the observational study design cannot eliminate the influence of residual confounders. Secondly, the study sample was based on middle-aged and elderly Chinese individuals, and the results may not be fully applicable to other races and age groups. Finally, LDL-C and HDL-C levels were measured only at baseline, preventing the assessment of their dynamic changes during follow-up with respect to hypertension risk.

## Conclusion

In conclusion, this study revealed a significant association between LHR and hypertension risk, which was particularly evident in specific subgroups and at different follow-up time points. Our findings emphasize the importance of monitoring and managing the LHR in middle-aged and elderly individuals for the prevention and control of hypertension. These findings provide a scientific basis for formulating personalized hypertension prevention strategies. Further research on the impact of different intervention measures on the LHR and hypertension risk is of significant public health importance.

## Data Availability

The original contributions presented in the study are included in the article/supplementary material. Further inquiries can be directed to the corresponding authors.
